# Fifteen-minute recess freestyle rope skipping enhances classroom attention in junior high school students in the context of healthy school development: mechanisms and implications

**DOI:** 10.3389/fpsyg.2026.1906224

**Published:** 2026-09-18

**Authors:** Jianmin Li, Xiaoting Chen, Wei Cen, Chao Liu

**Affiliations:** 1 College of Physical Education, Hefei University of Technology, Hefei, China; 2 College of Physical Education, Yangzhou University, Yangzhou, China

**Keywords:** 15-min recess, classroom academic emotions, classroom attention, freestyle rope skipping, healthy school development

## Abstract

In the context of healthy school development and the policy mandating 15-min recess physical activity for middle school students, this study employed a longitudinal quasi-experimental design to investigate the effects of 15-min recess freestyle rope skipping on middle school students' classroom attention and emotional indicators. Based on grade level and activity format (individual practice vs. group cooperation), a total of 160 students, 80 boys and 80 girls, aged 13.9 to 14.5 years, from grades 8 and 9 at a middle school in Shijiazhuang were selected and divided into four groups. A 16-week intervention involving individual vs. group freestyle rope skipping for 15 min per session was conducted. Students' recess immediate emotions, classroom academic emotions, and classroom attention were measured using validated self-report scales, specifically the Exercise-Induced Mood Scale, the Classroom Academic Emotion Scale, and the Classroom Attention Scale, at three time points (pre-intervention, mid-intervention, and post-intervention). Data were analyzed using repeated-measures ANOVA, correlation analysis, and mediation analysis. The results indicated that classroom attention significantly increased from 65.54 (pre-intervention) to 74.68 (post-intervention) across all groups. Classroom academic emotions and recess immediate emotions also exhibited significant changes over time, with significant time × group interaction effects, suggesting that the trajectories of change differed by grade level and activity format. Correlation analysis revealed that improvements in recess immediate emotions and classroom academic emotions were significantly positively correlated with increases in classroom attention, with the strongest correlation found between classroom academic emotions and classroom attention changes (*r* = 0.450, *p* < 0.001). Mediation analysis showed that classroom academic emotions significantly mediated the relationship between activity format and changes in classroom attention (indirect effect = 3.098, 95% CI [1.179, 5.525]), whereas the chain mediation pathway (recess immediate emotions → classroom academic emotions → classroom attention) was not significant. This study provides evidence that 15-min recess freestyle rope skipping is associated with improvements in middle school students' classroom attention and improve their emotional states, with the intervention effects moderated by grade level and activity format. This study provides empirical evidence for the fields of physical activity, emotion, and attention, and offers practical guidance for optimizing recess physical activities and promoting healthy school development in secondary schools.

## 1 Introduction

Schools bear the dual mission of imparting knowledge and promoting the physical and mental wellbeing of the next generation; they are the primary setting for fostering the healthy development of young people. In its *Global Framework for School Health Promotion*, the World Health Organization states that schools should serve as key settings for promoting the physical and mental wellbeing, cognitive development, and academic success of young people (Langford et al., 2015). Notably, regular physical activity enhances executive functions such as working memory, inhibitory control, and cognitive flexibility by promoting neuroplasticity and prefrontal cortex function (Stillman et al., 2020), and a recent meta-analysis confirms that physical exercise significantly improves these cognitive domains in adolescents with aerobic exercise showing particularly strong effects (Liu et al., 2025). The organization's *Guidelines on Physical Activity for Children and Adolescents* also emphasize that sufficient, high-quality daily physical activity is essential for improving mood, enhancing attention, and boosting learning outcomes (Chaput et al., 2020). The Chinese Ministry of Education's *Guiding Opinions on Comprehensively Promoting the Construction of Healthy Schools* similarly stipulates that primary and secondary school students must engage in at least 2 h of comprehensive physical activity daily, implement 15-min recess periods, and uphold the educational philosophy of health first (Ministry of Education of the People's Republic of China, 2026). However, the current mainstream school education model suffers from certain structural imbalances. It places excessive emphasis on sedentary, quiet academic instruction while neglecting the value of non-instructional time for student recovery and development, gradually creating an unhealthy norm (Li et al., 2024). Daily recess periods should serve as a golden window for cognitive reset—a time for psychological recovery, emotional regulation, and social interaction (Ramstetter et al., 2010). Yet, due to safety concerns, limited facilities, and the pressure of an academics-first mindset, these periods often devolve into passive rest or low-impact activities (Massey et al., 2020). As a result, students enter the next class burdened by accumulated fatigue and negative emotions, which directly impacts their concentration and learning outcomes in the classroom (Mazzoli et al., 2021). Therefore, a practical challenge arises: how to make effective use of the 15-min recess—a brief, frequent, and easily implementable resource—to design interventions that both capture students' interest and tangibly improve their focus in subsequent classes.

Against this backdrop, creative jump rope—with its rhythm, coordination, and fun, coupled with its low cost and high adaptability—is regarded as a relatively ideal option for filling the gap in high-quality recess activities (Wang et al., 2024). However, current research still has two key shortcomings, which are the core issues this study aims to address.

First, there is a significant lack of empirical research on the effectiveness of freestyle rope skipping activities in enhancing classroom attention during the specific context of the 15-min recess. Most existing studies on how physical activity improves attention have focused on short-term activities within the classroom, physical education classes, or specialized training sessions. Notably, 20-min bouts of moderate-to-vigorous physical activity have been shown to significantly enhance attention and reduce hyperactivity in children with ADHD (Pontifex et al., 2013; Suarez-Manzano et al., 2018), yet the current 15-min recess promoted in China falls short of this duration. Accumulating evidence indicates that school-based active breaks yield positive effects on students' on-task classroom behavior and selective attention, whereas evidence linking them to broader cognitive functions and academic achievement remains equivocal (Infantes-Paniagua et al., 2021; Vidal-Conti et al., 2026). The duration of these interventions, the intensity of the exercises, and the specific environmental conditions differ markedly from the natural setting of the 15-min recess currently being promoted in China (Janssen et al., 2014). Can middle school students achieve a consistent and measurable improvement in classroom attention through creative jump rope during these standardized, routine, and self-organized. Short recesses, though effects may vary by grade level and activity format (e.g., individual vs. group), and this variation remains insufficiently explored in current research (Liu et al., 2024; Woodard and Chen, 2024). As a result, while there is significant policy advocacy, there is a scarcity of empirical research that can effectively inform practical implementation.

Second, the psychological mechanisms by which creative jump rope activities affect classroom attention remain unclear, creating a black box phenomenon (Stillman et al., 2020). Neuroscience research has shown that a single session of moderate-intensity acute exercise can help optimize executive function by increasing cerebral blood flow and promoting the release of dopamine and brain-derived neurotrophic factor (Puneet et al., 2023). However, neurotransmitter changes are transient and cannot fully explain how physiological responses from a brief physical activity translate into sustained cognitive engagement across the temporal and contextual gap between recess and classroom instruction (Hashimoto and Ogoh, 2025). This limitation suggests that emotional processes may serve as a complementary mechanism, as emotions can persist across time and contexts, bridging physical activity experiences and subsequent classroom attention (Chang et al., 2025). Contemporary educational psychology posits that emotions serve as a crucial mediator linking the external environment to learning outcomes (Simonton and Garn, 2019). Creative jump rope activities during recess, particularly when practiced in group settings, may first elicit positive immediate emotional experiences in students, such as vitality and a sense of engagement (Thøgersen-Ntoumani et al., 2015). According to the Broaden-and-Build theory, positive emotions can temporarily broaden the scope of attention and cognitive resources (Chang et al., 2019); further, according to Control-Value theory, this refreshed emotional state transfers to the classroom, influencing students' subjective evaluations of learning tasks, thereby eliciting specific academic emotions, defined as affective reactions to academic tasks or experiences (Pekrun, 2006), such as interest, pride, or anxiety, in the classroom, such as generating interest and pride while reducing anxiety, which in turn drive changes in classroom attention. Based on this, the present study proposes a chain-like pathway hypothesis: creative jump rope activities during recess → immediate improvement in emotional state during recess → optimization of academic emotions in the classroom → enhanced classroom attention. This may represent a core psychological mechanism for understanding why 15 min of physical activity can enhance 45 min of learning (Osborne and Egan, 2023). However, this chain-like mediational pathway has not yet been empirically tested longitudinally in real-world recess settings.

Based on the aforementioned theoretical and empirical gaps, this study examines the core question of the effects of 15-min freestyle rope skipping during recess on middle school students'classroom attention and its underlying psychological mechanisms. The specific research hypotheses are as follows:

H1 (promotion effect hypothesis): using the pre-test data of each group as the within-group baseline, after 16 weeks of 15-min freestyle rope skipping during recess, middle school students' classroom attention, recess immediate emotions, and classroom academic emotions will all show significant longitudinal improvements, thereby validating that freestyle rope skipping has an overall promotional effect.

H2 (differential effect hypothesis): there will be significant differences in the changes across the three aforementioned indicators among students of different grade levels and between different activity organization formats (individual practice vs. group cooperation). This hypothesis is used to explore the boundary conditions under which the promotional effect occurs.

H3 (mechanism hypothesis—chain mediation): changes in recess immediate emotions and classroom academic emotions act as chain mediators in the relationship between activity organization format and changes in classroom attention. Specifically, group-based freestyle rope skipping indirectly enhances classroom attention by sequentially improving recess immediate emotions and classroom academic emotions, thereby revealing the psychological pathways underlying the promotional effect.

## 2 Method and materials

### 2.1 Participants

This study selected eighth- and ninth-grade students from a middle school in Shijiazhuang, Hebei Province, as the research subjects. Using cluster sampling, two classes from each grade were selected as experimental groups. Based on the research design and sample size estimation requirements, and taking into account the attrition rate typical of longitudinal tracking studies and the basic sample size needs for chain mediation analysis, 194 in-school middle school students were initially included. To ensure the representativeness of the participants and the validity of the data, this study strictly followed the *National Student Physical Fitness Standards* (2014 Edition) and the *Hebei Province Junior High School Academic Proficiency Test Standards for Physical Education and Health* to conduct multidimensional screenings of physical morphology, function, and athletic ability. Students with special athletic talents, those with congenital diseases, those failing to meet physical fitness standards, and those with abnormal daily activity levels, defined as extreme patterns of being chronically sedentary or excessively active relative to typical adolescent norms, were excluded, resulting in a final sample of 160 students who completed the pre-test, mid-test, and post-test-−80 boys and 80 girls. Ethical approval for this study was obtained from the Ethics Committee of Medical School of Yangzhou University. Written informed consent was provided by the participants' legal guardians prior to enrollment.

The study employed a 2 (grade: 8th grade/9th grade) × 2 (intervention type: individual/group) factorial design, comprising four groups: Grade 8 Individual Freestyle Rope Skipping Experimental Group (Group A), Grade 9 Individual Freestyle Rope Skipping Experimental Group (Group B), Grade 8 Group Freestyle Rope Skipping Experimental Group (Group C), and Grade 9 Group Freestyle Rope Skipping Experimental Group (Group D), with 40 students in each group. Results of the baseline homogeneity test showed no statistically significant differences among the four groups in demographic and physical fitness indicators such as age, height, weight, BMI, lung capacity, endurance run, 1-min jump rope, and daily exercise duration (*P* > 0.05). The balanced baseline levels made the groups comparable, providing a reliable foundation for the subsequent experimental intervention.

### 2.2 Study design

The study was conducted at a secondary school in Shijiazhuang using a longitudinal quasi-experimental design, with pre-test data from each group serving as the intra-group baseline. The intervention period ran from September 2025 to January 2026, examining changes in students' classroom attention, recess immediate emotions, and classroom academic emotions during 15-min freestyle rope skipping activities held during recess over a 16-week period, conducted twice daily, specifically during the T1 morning recess and the T3 afternoon recess. The study included three measurement time points: the pre-test 1 week before the official start of the activity, the mid-test after the completion of the 8th week of the activity, and the post-test after the completion of the 16th week of the activity. The study design was a 3 (Time: Pre-test, Mid-test, Post-test) × 2 (Grade: 8th grade, 9th grade) × 2 (Activity Format: individual freestyle rope skipping, group freestyle rope skipping) mixed design, with time as the within-subjects factor and grade level and activity format as between-subjects factors.

Grouping was based on students' grade level and the organizational format of the recess freestyle rope skipping activities, not on indicators of classroom attention or emotional state. During the pre-test phase, baseline data on students' demographic information, daily physical activity levels, and core psychological indicators were collected to assess the comparability of the starting points across groups.

To minimize the impact of the jump rope intervention on students' daily routines, such as mealtimes and dismissal, this study selected two time periods for the intervention (T1 and T3), as shown in Table 1.

**Table 1:** Distribution of 15-min recess activity periods at Shijiazhuang No. 21 Middle School.

Teaching time (before)	Short recess	Teaching time (after)
8:00–8:45	T1(Intervention)	9:00–9:45
10:15–11:00	T2	11:15–12:00
14:00–14:45	T3(Intervention)	15:00–15:45
15:00–15:45	T4	16:00–16:45

Recess periods are scheduled during two time slots: T1 and T3. T1 occurs after the first class of the morning, and T3 occurs after the first class of the afternoon. Both time slots fall within the break window between the completion of one class and the start of the next academic class, making it easier to observe the relationship between physical activity during recess and students' subsequent classroom performance. T2 falls near the latter part of the morning classes and the pre-lunch period, while T4 occurs near the end of the school day. These times involve numerous factors—such as students' physiological needs, preparations for leaving school, completing schoolwork, and accumulated fatigue—which are likely to introduce additional interference. Based on the stability of the school schedule, the safety of activity organization, and the feasibility of subsequent classroom measurements, this study selected T1 and T3 as the fixed activity periods.

Each recess activity lasted a total of 15 min; after deducting necessary time for restroom breaks, assembly, equipment preparation, and returning to the classroom, the effective duration of the freestyle rope skipping activity was approximately 10 min. Individual freestyle rope skipping focuses on individual movement coordination, rhythm control, and the experience of continuous performance; group freestyle rope skipping emphasizes peer cooperation, rhythmic coordination, and the experience of collective participation. Both activity formats are consistent in terms of duration, venue arrangement, musical rhythm, and safety management to minimize the influence of non-target factors. To ensure students' participation throughout the activity, teachers with research experience, along with subject teachers, conducted real-time visual monitoring and recorded instances of non-participation.

Variables related to instruction were managed through process control. For experimental classes within the same grade level, efforts were made to maintain the same subject teachers, a similar teaching pace, comparable homework loads, and a stable course schedule. The school's multi-departmental coordination mechanism is outlined in Table 2. Relevant teachers were briefed in advance, and the vice principal for academic affairs was responsible for promoting the full implementation of the 15-min recess exercise program, coordinating with teachers in the experimental classes to adjust their schedules, and ensuring that the 15-min recess activities did not conflict with the teaching plan. The physical education department is responsible for the actual implementation of the activity plan, controlling the musical rhythm, and guiding students to quickly engage in the activities. Homeroom teachers and subject teachers ensured students arrive on time and informed them to wear appropriate athletic attire daily. A significant limitation of the study is the absence of any measure of the intensity or quality of students' engagement during the intervention. Although teachers with research experience, along with subject teachers (Table 2), conducted real-time visual monitoring and recorded instances of non-participation, this method only confirms physical presence and basic compliance, not the depth or quality of student involvement.

**Table 2:** Personnel and responsibilities involved.

Personnel and responsibilities involved	Responsibilities
Vice principal for academic affairs	Coordinate teaching time and activity time
One rotating PE teacher per teaching week	Organize activities safely; control the music
Two PE teachers	Manage space arrangement; correct movement techniques
One PE teacher	Monitor students' physical condition during exercise
Two teachers with research experience	Distribute and collect questionnaires; assist with data collection and organization
Subject teachers of experimental classes	Observe dismissal time; remind students to bring jump ropes when leaving the classroom
Homeroom teachers of experimental classes	Instruct students to wear athletic shoes; conduct daily health monitoring and reporting

Assessment days were scheduled on regular school days, avoiding periods with major exams, school-wide events, and frequent class rescheduling. Classes following T1 and T3 sessions were regular academic classes; assessments were conducted immediately after these classes concluded. A 4-week pilot study was conducted prior to the formal experiment to evaluate the difficulty of the freestyle rope skipping movements, activity pace, and exercise intensity at different stages. The pilot study recorded students' heart rate changes, movement performance, and subjective feedback, and the formal intervention plan was adjusted accordingly.

A non-equivalent control group was not included in the present design due to practical constraints in the school setting. The participating school declined to allocate additional classes as a no-intervention control group, citing concerns that students in control classes might perceive inequity in access to the physical activity program during the 15-min recess period, which could potentially affect classroom order and morale. Furthermore, the 16-week intervention coincided with the regular academic calendar, and the school administration was unwilling to disrupt the established class schedules for a controlled trial. Under these circumstances, a longitudinal quasi-experimental design with each group serving as its own baseline control was adopted as a feasible alternative.

### 2.3 Measurement instruments

This study employed standardized scales frequently used in existing research to measure recess immediate emotions, classroom academic emotions, and classroom attention, specifically the Exercise-Induced Mood Scale, the Classroom Academic Emotion Scale, and the Classroom Attention Scale. The selection of scales took into account their relevance to the research topic, suitability for junior high school students, and practicality for on-site administration. While the structural fit of different scales within the sample may vary, the interpretation of results will focus primarily on longitudinal changes in total scores and key dimensions; measurement results will not be extrapolated to represent stable personality traits or general levels of mental health. It is important to note that the instruments employed capture state-level changes in emotion and attention during the intervention period; therefore, findings should not be extrapolated to represent stable personality traits or general mental health levels, which require trait-based assessments.

Measurements were conducted intermittently throughout the experiment to track changes in students' conditions following the intervention. These assessments were administered at the three time points specified in the study design. On each assessment day, immediately following a 15-min freestyle rope skipping activity during a designated recess, the Exercise-Induced Mood Inventory was distributed and collected; subsequently, during the immediately following academic class break, the Classroom Academic Emotion Questionnaire and the Classroom Attention Scale were distributed and collected. All questionnaires were distributed on a class-by-class basis. Before distribution, standardized instructions were read aloud to explain the anonymity and confidentiality of the questionnaires, as well as the response requirements, emphasizing that participants should answer based on their current genuine feelings. All questionnaires were distributed and collected on the spot. A total of 160 copies of each of the three questionnaires were distributed. After the collected paper questionnaires were sorted and numbered, the researcher entered the data into Excel to create a database. The data were labeled according to experimental groups (Group A, Group B, Group C, and Group D) and measurement time, and consistency checks and outlier screening were performed.

#### 2.3.1 Adolescent daily physical activity questionnaire

This questionnaire was designed to assess participants' total energy expenditure from moderate- and vigorous-intensity physical activity over the past week, as well as the time spent on sedentary activities such as homework, watching television, and using computers on school days and rest days (Li et al., 2011). This was done to ensure there were no significant differences among groups at baseline and to prevent pre-existing activity habits from interfering with the effectiveness of the 15-min recess freestyle rope skipping intervention. Regarding reliability, the correlation coefficients for energy expenditure at different intensities ranged from 0.81 to 0.88, with intraclass correlation coefficient (ICC) values of 0.82–0.89; for sedentary time, the correlation coefficients ranged from 0.82 to 0.85, with ICC values of 0.82–0.84. Regarding validity, the correlation coefficients with the criterion measure for energy expenditure ranged from 0.31 to 0.48, indicating high consistency. The aforementioned reliability and validity indicators demonstrate that the scale possesses good measurement stability and criterion-related validity, making it a suitable tool for baseline screening in this study.

#### 2.3.2 Exercise-induced mood scale

This scale was used to measure participants' immediate emotional states following short recess periods (Zhang, 2010). The questionnaire consisted of four subscales—vigor, mental calmness, physical fatigue, and active engagement—with three items per subscale, for a total of 12 items. The scoring range for each subscale was 0–15 points; higher scores indicated more pronounced energy restoration, greater mental calmness, stronger physical fatigue, and higher active engagement, respectively. Regarding reliability, the Cronbach's α for internal consistency of the total scale and each subscale ranged from 0.78 to 0.86. In terms of construct validity, the primary fit indices were CFI = 0.90, TLI = 0.91, RMSEA = 0.05. All fit indices met psychometric recommendations, indicating that the scale was suitable for use in this study.

#### 2.3.3 Classroom attention scale

This scale was used to assess students' attention levels in regular classroom settings (Ramos and Melo, 2019). It comprised four subscales—stimulus filtering, sensory control, competitive selection, and working memory—with a total of 19 items. Cronbach's α for the total scale and each subscale ranged from 0.83 to 0.88. Structural validity indices were CFI = 0.95, TLI = 0.93, and RMSEA = 0.06. All fit indices met psychometric recommendations, indicating that the scale was suitable for use in this study.

#### 2.3.4 Classroom academic emotion scale

This scale was used to measure the academic emotional states of junior high school students during classroom learning (Ren et al., 2022). It consisted of four subscales—positive high-arousal emotions, positive low-arousal emotions, negative high-arousal emotions, and negative low-arousal emotions—and contained a total of 45 items. Regarding reliability, the internal consistency coefficient for the total scale was 0.789, and the alpha coefficients for the four subscales all ranged between 0.7 and 0.8. In terms of construct validity, the primary fit indices were CFI = 0.96, TLI = 0.95, and RMSEA = 0.08. All fit indices met the recommended psychometric standards and were suitable for use in this study.

### 2.4 Statistical analysis

Data collected before, during, and after the experiment were entered into Excel in a standardized format, categorized, and preprocessed to identify and correct errors and exclude invalid samples. Confirmatory factor analysis was conducted using AMOS 28.0 to validate the validity of the measurement instruments, and statistical analysis was performed using SPSS 26.0. Results for all observed indicators were presented as mean ± standard deviation (*M* ± *SD*). The statistical significance level was set at α = 0.05, where *P* < 0.05 indicated a significant difference, *P* < 0.01 indicated a highly significant difference, and *P* > 0.05 indicated no significant difference.

An independent samples *t*-test was used to assess the homogeneity of demographic variables and daily physical activity levels across different grade levels and groups. Considering the differences in group composition across grade levels and intervention types, statistical analyses for longitudinal comparisons within groups and cross-stage comparisons between groups were conducted using mixed-design repeated measures ANOVA, sphericity tests, Greenhouse-Geisser corrections, Bonferroni *post-hoc* comparisons, change score correlation analyses. Furthermore, to examine whether changes in classroom academic emotions and recess immediate emotions mediated the relationship between activity format and changes in classroom attention, Bootstrap mediation tests were additionally conducted.

Change scores (post-test minus pre-test) were used in the mediation analysis to capture intra-individual changes over the intervention period. This approach is consistent with recommendations for analyzing intervention effects in longitudinal quasi-experimental designs (Cronbach and Furby, 1970). However, it should be noted that mediation analysis with change scores does not establish causal precedence among the mediators, as all change scores were derived from the same two time points. The results are therefore interpreted as statistical associations consistent with the hypothesized pathways rather than definitive evidence of causal sequencing (Maxwell and Cole, 2007). Activity format was dummy-coded (0 = individual, 1 = group) for all mediation analyses.

## 3 Results

Prior to evaluating the intervention effects, the baseline comparability of the four experimental groups was examined. Demographic characteristics and daily exercise duration were analyzed across grade levels to ascertain whether any subsequent changes in the outcome variables could be attributed to the intervention rather than to pre-existing group differences.

### 3.1 Demographic characteristics of participants across groups

As shown in Table 3, to account for differences in age, height, weight, and daily exercise duration among Experimental Groups A, B, C, and D, homogeneity tests were conducted using independent samples *t*-tests, with participants grouped by 8th and 9th grade. The results showed that for the eighth-grade group, there were no statistically significant differences in age (*t* = −0.787, *P* = 0.435), height (*t* = 0.493, *P* = 0.623), weight (*t* = 0.279, *P* = 0.781), or daily exercise duration (*t* = 0.110, *P* = 0.912); in the ninth-grade group, there were no statistically significant differences in age (*t* = −1.595, *P* = 0.115), height (*t* = 0.188, *P* = 0.851), weight (*t* = 0.164, *P* = 0.870), or daily exercise duration (*t*= 0.128, *P* = 0.898).

**Table 3:** Demographic variables by group.

Variable	Grade 8		Grade 9	
	Individual	Team	Individual	Team
Age (years)	13.88 ± 0.34	13.95 ± 0.50	14.33 ± 0.47	14.50 ± 0.51
Height (cm)	170.25 ± 2.99	169.93 ± 2.91	175.10 ± 2.91	174.98 ± 3.02
Weight (kg)	56.10 ± 2.01	55.98 ± 1.99	58.05 ± 2.01	57.98 ± 2.08
Daily exercise duration (min)	40.18 ± 6.11	40.03 ± 6.05	39.90 ± 6.06	39.73 ± 6.15

### 3.2 Analysis of variance in results across pre-test, mid-test, and post-test

The longitudinal analysis sample comprised the four groups described in the Method section, with each group consisting of 40 students. The three scales—classroom attention, classroom academic emotion, and recess immediate emotions—maintained a sample size of 40 students per group across the pre-test, mid-test, and post-test phases, ensuring consistency in the longitudinal analysis sample size. Among the four pre-test raw item tables, Group B had 42 students in the raw records; the longitudinal statistical analysis was based on the 160 students who had complete data for all three measurements in the summary table of psychological indicators.

Common method bias was assessed using Harman's single-factor test on complete cases with all pre-test items. The results showed that 24 factors with eigenvalues greater than 1 were extracted from 84 complete cases and 76 items; the first common factor explained 9.81% of the variance, which is below the 40% cutoff criterion. This result indicates that no single factor dominated the majority of the variance in the pre-test items. Since this test was based solely on the pre-test raw items, and given that the pre-test raw items contained missing data, the results serve only as a supplementary assessment of the risk of common method bias.

Table 4 shows that the total scores for classroom attention exhibited different trends across the four groups. Group A increased from 64.05 on the pre-test to 66.60 on the mid-test, then decreased to 60.12 on the post-test; Group B increased from 67.12 on the pre-test to 88.80 on the post-test; Group C increased from 65.78 on the pre-test to 82.30 on the mid-test, with a post-test score of 79.25; Group D increased from 65.20 on the pre-test to 70.55 on the post-test. This table only indicates that the direction of change in the mean scores differs across groups; it cannot be used to determine statistical significance.

**Table 4:** Descriptive statistics of recess immediate emotions, classroom academic emotions, and classroom attention (*M*, SD).

Indicator	Group	Pre-test	Mid-test	Post-test
Classroom attention	A	64.05 ± 6.73	66.60 ± 7.75	60.12 ± 5.36
	B	67.12 ± 7.97	72.72 ± 7.18	88.80 ± 6.31
	C	65.78 ± 8.30	82.30 ± 9.30	79.25 ± 8.70
	D	65.20 ± 7.94	66.80 ± 8.75	70.55 ± 11.30
Classroom academic emotions	A	142.95 ± 16.54	156.57 ± 13.21	153.95 ± 8.36
	B	145.25 ± 15.11	168.85 ± 9.39	185.82 ± 9.32
	C	142.68 ± 13.23	165.78 ± 8.49	185.35 ± 18.05
	D	141.45 ± 16.06	162.85 ± 13.33	179.20 ± 25.23
Recess immediate emotions	A	26.05 ± 5.21	26.32 ± 4.62	26.48 ± 2.08
	B	25.35 ± 5.53	26.65 ± 5.69	32.48 ± 4.14
	C	25.70 ± 5.70	30.85 ± 4.10	31.45 ± 5.56
	D	25.70 ± 5.82	26.85 ± 5.49	32.88 ± 3.98

The total scores for classroom academic emotions were higher at the post-test than at the pre-test in all four groups. Group A increased from 142.95 to 153.95, Group B from 145.25 to 185.82, Group C from 142.68 to 185.35, and Group D from 141.45 to 179.20. Regarding the total scores for recess immediate emotions, Groups B, C, and D showed a greater increase from pre-test to post-test, while Group A changed from 26.05 to 26.48, representing a smaller change. This table provides a descriptive foundation for the subsequent repeated-measures analysis of variance.

Of particular note, Group A (eighth-grade individual format) exhibited a distinct pattern: classroom attention scores increased from pre-test to mid-test but then declined substantially at post-test, falling below the pre-test level. This pattern was not observed in the other three groups, warranting further examination in the discussion.

Table 5 shows that the Mauchly sphericity test for the total scores of classroom attention, classroom academic emotions, and recess immediate emotions all reached statistical significance (*P* < 0.001), indicating that none of the three total scores met the sphericity assumption. The GGε for the total classroom attention score was 0.720, for the total classroom academic emotions score was 0.526, and for the total recess immediate emotions score was 0.609. Subsequent repeated-measures ANOVAs involving time effects were interpreted using the results of the Greenhouse-Geisser correction.

**Table 5:** Mauchly's Test of sphericity and Greenhouse-Geisser correction parameters for total scores of three core indicators.

Indicator	*N*	Mauchly's W	χ^2^	df	*P*	GGε
Classroom attention	160	0.611	77.882	2	<0.001	0.720
Classroom academic emotions	160	0.101	362.829	2	<0.001	0.526
Recess immediate emotions	160	0.357	162.760	2	<0.001	0.609

GGε, Greenhouse-Geisser epsilon.

A *p*-value less than 0.05 indicates violation of the sphericity assumption.

Table 6 shows that the main effects of group and time, as well as the time × group interaction effect, for the total classroom attention score were all significant, with all *P* < 0.001. The ηp^2^ for the time × group interaction effect was 0.370, indicating that the trajectories of changes in classroom attention differed across the four groups. Combined with Table 4, it can be seen that Group A's post-test scores were lower than their pre-test scores, while Groups B, C, and D's post-test scores were higher than their pre-test scores; thus, changes in classroom attention cannot be generalized as a consistent improvement across all groups.

**Table 6:** Time × group mixed repeated-measures ANOVA for total scores of three core indicators.

Indicator	Effect	*F*	df1	df2	*P*	*ηp* ^2^
Classroom attention	Group	57.863	3	156	<0.001	0.527
	Time	61.276	2	312	<0.001	0.282
	Time × Group	30.589	6	312	<0.001	0.370
Classroom academic emotions	Group	16.142	3	156	<0.001	0.237
	Time	305.388	2	312	<0.001	0.662
	Time × Group	15.835	6	312	<0.001	0.233
Recess immediate emotions	Group	11.957	3	156	<0.001	0.187
	Time	111.910	2	312	<0.001	0.418
	Time × Group	17.652	6	312	<0.001	0.253

ηp^2^, partial eta squared.

Time effects were interpreted using the Greenhouse-Geisser correction.

The main effects of group and time, as well as the time × group interaction effect, for the total classroom academic emotion score were all significant (*P* < 0.001). The ηp^2^ for the main effect of time was 0.662, indicating a large effect size. As shown in Table 4, the post-test total classroom academic emotion scores for all four groups were higher than the pre-test scores, with differences among groups reflected in the varying degrees of improvement.

The main effects of group and time, as well as the time × group interaction effect, for the total score of recess immediate emotions all reached statistical significance (*P* < 0.001). The η*p*^2^ for the main effect of time was 0.418, and the η*p*^2^ for the time × group interaction effect was 0.253. As shown in Table 4, Groups B, C, and D showed a pronounced increase in post-test scores compared to pre-test scores, while Group A exhibited a smaller change.

Table 7 shows that the time effects for all four dimensions of classroom attention were significant (all *P* < 0.001); the time × group interaction effects were also significant (all *P* < 0.001). Specifically, the time × group interaction effect η*p*^2^ was 0.225 for competitive selection and 0.203 for working memory, which were higher than those for stimulus filtering and sensory control. This indicates that group differences in changes in classroom attention were primarily reflected in the two dimensions of competitive selection and working memory.

**Table 7:** Time × group mixed repeated-measures ANOVA for each dimension.

Scale	Indicator	Time effect *F*	Time effect *p*	Time × group *F*	Time × group p	Time × group ηp^2^
Classroom attention	Stimulus filtering	18.312	<0.001	7.445	<0.001	0.125
	Sensory control	31.354	<0.001	6.112	<0.001	0.105
	Competitive selection	25.319	<0.001	15.062	<0.001	0.225
	Working memory	20.034	<0.001	13.242	<0.001	0.203
Classroom academic emotions	Positive high-arousal	294.271	<0.001	38.221	<0.001	0.424
	Positive low-arousal	153.618	<0.001	3.578	0.002	0.064
	Negative high-arousal	76.935	<0.001	4.461	<0.001	0.079
	Negative low-arousal	115.738	<0.001	4.754	<0.001	0.084
Recess immediate emotions	Vigor	70.235	<0.001	4.871	<0.001	0.086
	Mental calmness	49.448	<0.001	4.899	<0.001	0.086
	Physical fatigue	4.123	0.017	4.890	<0.001	0.086
	Active engagement	66.306	<0.001	10.593	<0.001	0.169

The time effects for all four dimensions of classroom academic emotion were significant (all *P* < 0.001). The time × group interaction effect for positive high arousal was *F* = 38.221, η*p*^2^ = 0.424, making it the dimension with the most pronounced group differences in this scale. The time × group interaction effects for positive low arousal, negative high arousal, and negative low arousal were also significant, indicating that each dimension of classroom academic emotion followed distinct trajectories of change over time and across groups.

The time effects for all four dimensions of recess immediate emotions were significant. The time effect for physiological fatigue was *F* = 4.123, *P* = 0.017, with an effect size η*p*^2^ = 0.026, which was lower than those for vitality, mental calmness, and active engagement. The time × group interaction effect for active engagement had an η*p*^2^ of 0.169, higher than the other three dimensions of recess immediate emotions, indicating that differences in activity engagement were more pronounced across groups.

Table 8 shows that significant differences were found in the total scores for classroom attention from the pre-test to the mid-test and from the pre-test to the post-test, with *P* < 0.001 for both; however, the difference from the mid-test to the post-test was not significant (*P* = 0.068). These results indicate that the overall improvement in classroom attention was primarily evident in the comparisons from the pre-test to the mid-test and from the pre-test to the post-test, while there was insufficient evidence of continued change after the mid-test.

**Table 8:** Bonferroni-corrected pairwise comparisons for total scores of three core indicators across three time points.

Indicator	Comparison	M (Pre)	M (Post)	Mean difference	Adjusted *p*
Classroom attention	Pre vs. Mid	65.54	72.11	6.57	<0.001
	Pre vs. Post	65.54	74.68	9.14	<0.001
	Mid vs. Post	72.11	74.68	2.58	0.068
Classroom academic emotions	Pre vs. Mid	143.08	163.51	20.43	<0.001
	Pre vs. Post	143.08	176.08	33.00	<0.001
	Mid vs. Post	163.51	176.08	12.57	<0.001
Recess immediate emotions	Pre vs. Mid	25.70	27.67	1.97	<0.001
	Pre vs. Post	25.70	30.82	5.12	<0.001
	Mid vs. Post	27.67	30.82	3.15	<0.001

Adjusted *p, p*-values corrected using the Bonferroni method.

Significant differences were observed in the total scores for classroom academic emotions across all three time points (*P* < 0.001 for all). The mean difference from pre-test to post-test was 33.00, higher than the 20.43 observed from pre-test to mid-test, indicating that classroom academic emotions continued to change throughout the 16-week program.

The total scores for recess immediate emotions were significant across all three time comparisons (all *P* < 0.001). The mean difference from pre-test to post-test was 5.12, and the mean difference from mid-test to post-test was 3.15, indicating that recess immediate emotions continued to change significantly during the latter half of the program.

Table 9 shows that, regarding the change in total classroom attention scores from the pre-test to the mid-test, the grade effect, the activity format effect, and the grade × activity format interaction effect were all significant. Regarding the change from the pre-test to the post-test, the grade effect and the grade × activity format interaction effect were significant, while the main effect of activity format was not significant (*P* = 0.741). These results indicate that long-term changes in classroom attention cannot be simply attributed to individual or group activity formats alone; the combined relationship between grade level and activity format is more critical.

**Table 9:** Supplemental ANOVA for change scores by grade × activity format.

Indicator	Comparison	Effect	*F*	*P*	*ηp* ^2^
Classroom attention	Mid–Pre	Grade	12.248	0.001	0.073
		Activity format	8.642	0.004	0.052
		Grade × activity format	28.063	<0.001	0.152
	Post–Pre	Grade	28.994	<0.001	0.157
		Activity format	0.110	0.741	0.001
		Grade × Activity Format	107.988	<0.001	0.409
Classroom academic emotions	Mid–Pre	Grade	10.401	0.002	0.063
		Activity format	8.039	0.005	0.049
		Grade × Activity Format	20.704	<0.001	0.117
	Post–Pre	Grade	12.759	<0.001	0.076
		Activity format	17.477	<0.001	0.101
		Grade × activity format	24.992	<0.001	0.138
Recess immediate emotions	Mid–Pre	Grade	9.558	0.002	0.058
		Activity format	24.109	<0.001	0.134
		Grade × activity format	27.268	<0.001	0.149
	Post–Pre	Grade	24.879	<0.001	0.138
		Activity format	10.888	0.001	0.065
		Grade × activity format	10.486	0.001	0.063

ηp^2^, partial eta squared.

For the changes in the total score for classroom academic emotions from the pre-test to the mid-test and from the pre-test to the post-test, the grade effect, activity format effect, and grade × activity format interaction effect all reached statistical significance. These results indicate that the magnitude of changes in classroom academic emotions is influenced by both grade level and activity organization format.

In the changes in the total score for recess immediate emotions from the pre-test to the mid-test and from the pre-test to the post-test, the grade effect, activity format effect, and grade × activity format interaction effect all reached statistical significance. These results indicate that changes in recess immediate emotions exhibit distinct differences across grade levels and activity formats. This table supports the hypothesis that students in different grade levels and with different activity formats exhibit distinct trajectories of change, but does not support the unidirectional conclusion that a particular grade level or activity format is necessarily superior.

### 3.3 Correlation analysis and exploratory mechanism testing

Table 10 shows that, at the post-test total score level, there were positive correlations among recess immediate emotions, classroom academic emotions, and classroom attention. Among these, the correlation between classroom academic emotions and classroom attention was the strongest, at *r* = 0.673; the correlation between recess immediate emotions and classroom attention was *r* = 0.392; and the correlation between recess immediate emotions and classroom academic emotions was *r* = 0.306.

**Table 10:** Correlations among core variables.

Type	Variable 1	Variable 2	*r*	*P*
Post-test	Recess emotions	Academic emotions	0.306	—
	Recess emotions	Attention	0.392	—
	Academic emotions	Attention	0.673	—
Post-Pre Change	Recess emotions change	Academic emotions change	0.220	0.005
	Recess emotions change	Attention change	0.277	<0.001
	Academic emotions change	Attention change	0.450	<0.001

Post-test correlations from correlation matrix.

Change-score correlations are Pearson's *r*.

Dash, not reported.

At the level of change, changes in recess immediate emotions were significantly positively correlated with changes in classroom academic emotions (*r* = 0.220, *P* = 0.005); changes in recess immediate emotions were significantly positively correlated with changes in classroom attention (*r* = 0.277, *P* < 0.001); changes in classroom academic emotions were significantly positively correlated with changes in classroom attention (*r* = 0.450, *P* < 0.001). This table indicates that changes in emotional states are synchronously associated with changes in classroom attention, with a stronger link observed between changes in classroom academic emotions and changes in classroom attention.

Table 11 shows that activity format significantly predicts changes in recess immediate emotions, with an estimate of 2.688 and a 95% confidence interval of [1.077, 4.280]; activity format also significantly predicts changes in classroom academic emotions, with an estimate of 13.472 and a 95% confidence interval of [6.096, 21.682]. Changes in classroom academic emotions significantly predict changes in classroom attention, with an estimate of 0.230 and a 95% confidence interval of [0.156, 0.302].

**Table 11:** Bootstrap exploratory mechanism test based on change scores.

Path or effect	Estimate	BootLLCI	BootULCI	Result
Activity format → recess emotions change	2.688	1.077	4.280	Significant
Activity format → academic emotions change	13.472	6.096	21.682	Significant
Recess emotions chang → academic emotions change	0.355	−0.420	1.125	Not significant
Recess emotions change → attention change	0.390	−0.000	0.765	Not significant
Academic emotions change → attention change	0.230	0.156	0.302	Significant
Activity format → attention change (direct effect)	−3.828	−7.748	0.064	Not significant
Activity format → recess emotions change → attention change	1.048	−0.005	2.486	Not significant
Activity format → academic emotions change → attention change	3.098	1.179	5.525	Significant
Activity format → recess emotions change → academic emotions chang → attention change	0.219	−0.291	0.705	Not significant
Total indirect effect	4.365	2.481	6.597	Significant

BootLLCI and BootULCI, lower and upper limits of the 95% Bootstrap confidence interval, respectively.

An effect is considered significant if the confidence interval does not contain zero.

The indirect effect of activity format on changes in classroom attention via changes in classroom academic emotions is significant, with an effect size of 3.098 and a 95% confidence interval of [1.179, 5.525]. The indirect effect of activity format on changes in classroom attention via changes in recess immediate emotions did not reach statistical significance, with the 95% confidence interval including zero. The chain path of recess immediate emotions → changes in classroom academic emotions → changes in classroom attention also did not reach statistical significance, with an effect size of 0.219 and a 95% confidence interval of [−0.291, 0.705].

For the mediation analysis, activity format was dummy-coded (0 = individual, 1 = group). Grade level was entered as a covariate. All change scores were computed as post-test minus pre-test values. Bootstrap resampling with 5,000 iterations was used to estimate 95% confidence intervals for indirect effects, using PROCESS macro for SPSS (Model 4 for simple mediation, Model 6 for serial mediation; Hayes, 2018).

These results should be interpreted as statistical associations based on change scores rather than causal pathways, as the mediation analysis was conducted with longitudinal observational data and does not establish temporal precedence among the mediators.

## 4 Discussion

### 4.1 Differential changes in classroom attention during recess freestyle rope skipping activities

This study used the pre-test data from each group as a baseline reference and employed a longitudinal quasi-experimental design to examine changes in classroom attention during 15-min recess freestyle rope skipping activities over a 16-week period. The results indicate a significant main effect of time on the total classroom attention score, and the time × group interaction effect also reached statistical significance. This suggests that classroom attention does not change in a single direction over the course of the activity, and that the trajectories of change differ among students across grade levels and activity formats. Descriptive results revealed divergent post-test trajectories across the four groups: Groups B, C, and D improved relative to baseline, while Group A declined. These patterns suggest that recess freestyle rope skipping is associated with changes in classroom attention, with the magnitude of these changes moderated by factors such as grade level, activity format, and classroom context.

Existing research provides theoretical support for these findings. Basso and Suzuki (2017) noted that acute physical activity can influence emotional, cognitive, neurophysiological, and neurochemical pathways, thereby potentially providing a foundation for improved attention control and learning readiness. A meta-analysis by Verburgh et al. (2014) also found a positive relationship between physical activity and executive function in children, adolescents, and young adults. Freestyle rope skipping combines rhythmicity, coordination, and the requirement for movement transitions; during the activity, students must continuously adjust their body posture, movement speed, and the rhythm of the rope. These processes are associated with competitive selection, working memory, and stimulus filtering in classroom attention (Chen et al., 2015).

The findings also suggest that the impact of recess activities on classroom attention should not be simplistically interpreted as the more exercise, the better or one particular form is necessarily superior. This is consistent with studies examining other forms of school-based physical activity, such as running or coordinative exercise, which have also found that the cognitive benefits of physical activity depend not only on intensity but also on the type and structure of the activity (e.g., Chen et al., 2015; Liu et al., 2025). Herold et al. (2019) noted that dose-response relationships are crucial in exercise-cognition research, as duration, intensity, form, and individual adaptability all influence cognitive outcomes. However, the current discussion about dose-response effects actually addresses intervention modalities rather than true dose-response relationships; this distinction should be clarified. In this study, the main effect of activity format on the post-test pre-test change in classroom attention did not reach statistical significance, while the grade × activity format interaction effect was significant, indicating that differences between individual and group activities cannot be explained independently of grade-level characteristics. This finding aligns with the perspective of Novak et al. (2025) regarding the differing effects of individual vs. group exercise on adolescents' physical activity levels, and suggests that recess activity design must take into account both activity format and students' developmental stage. However, given the absence of objective measures of engagement intensity, it is plausible that variations in the quality of student involvement, rather than the intervention format *per se*, contributed to the observed effects. This limitation should be considered when interpreting the current findings.

The decline in Group A's classroom attention scores from mid-test to post-test was concentrated in the working memory dimension, a cognitive domain directly influenced by motivational and emotional engagement (Lubans et al., 2016). Among the four groups, only Group A (eighth-grade individual format) exhibited this pattern. The data were re-examined for entry errors and no clerical anomalies were identified; assessment days were also scheduled to avoid major examinations and school events, which rules out these as confounding factors. The pilot study heart rate data indicated that exercise intensity was adequately controlled across groups, which rules out differential fatigue as an explanation. Individual rope skipping lacks sufficient novelty and social reinforcement to sustain engagement over a 16-week period among eighth-grade students. Group formats, by contrast, provide peer interaction and collective efficacy, which sustain motivation and cognitive engagement over extended interventions. This is supported by the finding that Group C (8th-grade group format) maintained higher post-test scores than Group A.

### 4.2 The relationship between changes in classroom academic emotions and changes in classroom attention is relatively stable

This study shows that there is a significant main effect of time on the total score of classroom academic emotions, and a significant time × group interaction effect. Correlation analysis of change scores revealed a significant positive correlation between changes in classroom academic emotions and changes in classroom attention, with a stronger correlation than that observed between changes in recess immediate emotions and changes in classroom attention. Mechanism tests based on change scores further indicated that changes in classroom academic emotions exhibited a significant indirect effect in the statistical model on the relationship between activity format and changes in classroom attention. These findings suggest that classroom academic emotions may serve as a relatively stable psychological link between the context of recess physical activity and subsequent classroom attention. Tamura et al. (2022) found that low-intensity physical activity can influence emotional and cognitive engagement in the classroom, which is consistent with the stable link between changes in classroom academic emotions and changes in classroom attention observed in this study.

Positive emotion theory can also explain this result. Fredrickson (2001) proposed that positive emotions have a broaden-and-build effect, capable of expanding an individual's cognitive and behavioral resources. A systematic review and meta-analysis by Li et al. (2022) demonstrated that physical activity has a positive impact on positive emotions in children and adolescents. If freestyle rope skipping activities during recess can provide a positive sense of participation, a sense of achievement, and a state of engagement in the classroom, they may help students maintain more positive classroom academic emotions in subsequent lessons. The systematic review by Rodriguez-Ayllon et al. (2019) also noted that there is a positive association between physical activity and mental health in children and adolescents. In light of the findings of this study, classroom academic emotions are not a secondary outcome but rather a key psychological variable for understanding changes in classroom attention.

### 4.3 Recess immediate emotions have change value, but chain mediation pathways should be interpreted with caution

The results of this study indicate that the total score for recess immediate emotions exhibits a significant main effect of time and a time × group interaction effect; dimensions such as vitality, mental calmness, physical fatigue, and active engagement also show varying degrees of change. These findings suggest that the 15-min freestyle rope skipping activity during recess can lead to changes in students' recess immediate emotions. Correlation analysis of the change values revealed a significant positive correlation between changes in recess immediate emotions and changes in classroom attention, indicating a synchronous relationship between immediate mood changes and subsequent classroom attention.

Simonton and Garn (2020) noted that negative emotions in physical education settings can predict student behavioral outcomes, suggesting that emotional experiences during physical activity influence subsequent behavioral performance. During the recess freestyle rope skipping activities, students may experience multiple states such as renewed vitality, physical fatigue, engagement, and emotional release; these immediate experiences may affect their readiness for the next class. Yan et al. (2024), in their study on the chain of relationships among physical activity, mental health, negative emotions, and self-efficacy among middle school students, also suggest that there are multi-step connections between physical activity and psychological states. Che et al. (2025), in their research on the chain mechanisms linking adolescents' physical activity, academic performance, self-regulation, and self-efficacy, similarly demonstrate that the relationship between physical activity and learning outcomes is often not a single-pathway process.

The hypothesized chain mediation pathway was not supported in the change-score model. The path from changes in recess immediate emotions to changes in classroom academic emotions did not reach statistical significance, and the confidence interval for the chain-mediated effect included zero. This indicates that although recess immediate emotions correlate with changes in classroom attention, their influence is not sequentially transmitted through classroom academic emotions. The translation of recess immediate emotions into classroom academic emotions may be moderated by classroom content, teacher organization, student academic pressure, and perceived task value (Li, 2021). In addition, the non-significant chain mediation likely reflects a methodological constraint: all variables were derived from the same post-pre change scores, which does not establish temporal ordering among the mediators. Testing a sequential pathway requires temporally separated measurements to preserve temporal precedence. Future studies should adopt multi-wave longitudinal designs to evaluate this dynamic pathway.

### 4.4 Differences in grade level and activity format manifest as differences in change trajectories

Supplementary analyses of change measures revealed that the magnitude of change in classroom attention, classroom academic emotions, and recess immediate emotions was influenced by grade level and activity format. Significant grade × activity format interaction effects were observed for some indicators. These results suggest that differences in grade level and activity format are not merely a matter of main effects, but rather manifest as differences in change trajectories under combined conditions. Bandura (1977) proposed that self-efficacy influences an individual's behavioral choices, effort, and persistence. Individual freestyle rope skipping places greater emphasis on mastering movements and self-regulation, whereas group freestyle rope skipping emphasizes peer cooperation and collaborative achievement. Differences among students of different grades in motor learning, self-efficacy, need for peer support, and classroom stress may lead to varying effects of activity formats. A study by Ren et al. (2020) on social support, physical activity, and exercise self-efficacy among Chinese adolescents indicated that social support influences physical activity through exercise self-efficacy, providing a reference for the relationship between peer support and self-efficacy in group activities.

Based on the findings of this study, group activities did not demonstrate a consistent advantage across all measures, nor were individual activities less effective in all groups. The design of freestyle rope skipping activities during recess should be adjusted based on students' grade level, motor skills, need for peer interaction, and academic stress. For younger students, the interaction and support provided by group activities may help sustain their interest in participation (Qi et al., 2024); for older students, the sense of self-control and the experience of successfully completing movements during individual activities may also be linked to improvements in classroom attention (Shen et al., 2022).

### 4.5 Limitations and future directions

Several limitations of this study should be acknowledged. First, the absence of a no-intervention control group limits the attribution of the observed changes solely to the freestyle rope skipping intervention. Maturation effects, history effects, or testing effects may have contributed to the observed improvements over the 16-week period. The longitudinal within-group design provides evidence of change relative to baseline, but it cannot rule out secular trends that may have affected all participants independently of the activity (Andrade, 2021). Second, the generalizability of the findings is constrained by the sample being drawn from a single school in Shijiazhuang, and the cluster sampling method limits the representativeness of the results. Third, the reliance on self-report questionnaires for emotional and attentional measurement introduces the potential for social desirability bias and common method variance, although Harman's single-factor test indicated that common method bias was not a substantial concern in this study.

Fourth, the decline in classroom attention observed in Group A from mid-test to post-test indicates that individual rope skipping may not be sustainable for eighth-grade students over a 16-week intervention. The absence of process measures, such as weekly engagement ratings or enjoyment checks, prevents a more precise identification of the mechanisms underlying this decline. Fifth, the mediation analysis using change scores tests statistical associations rather than causal pathways. All mediators and the outcome were derived from the same post-pre differences, which does not establish temporal precedence.

Future research should incorporate a randomized controlled trial design with a no-intervention control group to strengthen causal inferences regarding the effects of recess-based physical activity on classroom attention. The inclusion of objective measures, such as classroom observation of on-task behavior, actigraphy for physical activity monitoring, and physiological indicators like heart rate variability or salivary cortisol, would provide more robust and triangulated evidence for the psychological mechanisms identified in this study. Expanding the sample to multiple schools across different regions would enhance the external validity of the findings. For mediation analyses, future studies should adopt multi-wave longitudinal designs with temporally separated measurements to evaluate the dynamic pathways from physical activity to cognitive outcomes.

## 5 Conclusion

This study employed a longitudinal quasi-experimental design, using the pre-test data from each group as an intra-group baseline reference. This design allowed us to observe the dynamic changes in students' outcomes during 16 weeks of freestyle rope skipping activities during recess, as well as to compare the trajectories of change across different groups. The study yielded the following conclusions.

(1) Using the pre-test data of each group as the intra-group baseline reference, significant temporal changes were observed in middle school students' classroom attention, classroom academic emotions, and recess immediate emotions during the 16-week intervention. Significant group differences were found in changes in classroom attention, with Groups B, C, and D showing improvements while Group A declined from mid-test to post-test.

(2) Changes in classroom academic emotions were significantly positively correlated with changes in classroom attention. Exploratory mechanism analysis based on the magnitude of change indicated that changes in classroom academic emotions exerted a significant indirect effect on the relationship between activity format and changes in classroom attention, suggesting that classroom academic emotions may serve as a crucial psychological pathway linking the physical activity context during recess to subsequent classroom attention.

(3) There was a significant correlation between changes in recess immediate emotions and changes in classroom attention; however, the chain-mediated path from recess immediate emotions to classroom academic emotions to classroom attention did not reach statistical significance. The conditions under which recess immediate emotions translate into classroom academic emotions and classroom attention require further investigation in future studies.

The findings should be interpreted within the context of a longitudinal quasi-experimental design without an external control group. Future randomized controlled trials with objective measures and multi-wave mediation designs are needed to confirm the causal pathways suggested by this study.

## Data Availability

The raw data supporting the conclusions of this article will be made available by the authors, without undue reservation.
